# Influence of Material Selection on the Marginal Accuracy of CAD/CAM-Fabricated Metal- and All-Ceramic Single Crown Copings

**DOI:** 10.1155/2018/2143906

**Published:** 2018-03-22

**Authors:** Matthias Rödiger, Lea Schneider, Sven Rinke

**Affiliations:** ^1^Department of Prosthodontics, University Medical Center Goettingen, Robert-Koch-Str. 40, 37075 Goettingen, Germany; ^2^Private Practice, Geleitstr. 68, 63456 Hanau, Germany

## Abstract

This study evaluated the marginal accuracy of CAD/CAM-fabricated crown copings from four different materials within the same processing route. Twenty stone replicas of a metallic master die (prepared upper premolar) were scanned and divided into two groups. Group 1 (*n* = 10) was used for a pilot test to determine the design parameters for best marginal accuracy. Group 2 (*n* = 10) was used to fabricate 10 specimens from the following materials with one identical CAD/CAM system (GAMMA 202, Wissner GmbH, Goettingen, Germany): A = commercially pure (cp) titanium, B = cobalt-chromium alloy, C = yttria-stabilized zirconia (YSZ), and D = leucite-reinforced glass-ceramics. Copings from group 2 were evaluated for the mean marginal gap size (MeanMG) and average maximum marginal gap size (AMaxMG) with a light microscope in the “as-machined” state. The effect of the material on the marginal accuracy was analyzed by multiple pairwise comparisons (Mann–Whitney, *U*-test, *α* = 0.05, adjusted by Bonferroni-Holmes method). MeanMG values were as follows: A: 46.92 ± 23.12 *μ*m, B: 48.37 ± 29.72 *μ*m, C: 68.25 ± 28.54 *μ*m, and D: 58.73 ± 21.15 *μ*m. The differences in the MeanMG values proved to be significant for groups A/C (*p* = 0.0024), A/D (*p* = 0.008), and B/C (*p* = 0.0332). AMaxMG values (A: 91.54 ± 23.39 *μ*m, B: 96.86 ± 24.19 *μ*m, C: 120.66 ± 32.75 *μ*m, and D: 100.22 ± 10.83 *μ*m) revealed no significant differences. The material had a significant impact on the marginal accuracy of CAD/CAM-fabricated copings.

## 1. Introduction

Metal-ceramic and all-ceramic single crowns are accepted prosthetic treatment options with a good long-term performance that is documented in numerous clinical trials [1]. Traditionally, metal-ceramic and all-ceramic restorations require different fabrication techniques, for example, casting, heat-pressing, and slip-casting. Currently, metallic and all-ceramic crown restorations can be fabricated by using computer-aided design/computer-aided manufacturing (CAD/CAM) technology [2].

The most established CAD/CAM production technique is milling/grinding of metallic and all-ceramic materials. Nonprecious metal alloys (e.g., cobalt-chromium alloys) or commercially pure (cp) titanium have been used in dental CAD/CAM technology for more than two decades [3]. Due to suitable CAD/CAM technologies and the availability of high strength framework ceramics with an excellent biocompatibility (e.g., lithium-disilicate or yttria-stabilized zirconia (YSZ)), it is also possible to fabricate all-ceramic crowns with adequate fracture-resistance [3].

Therefore, a variety of metal and all-ceramic materials is available for crown fabrication in the digital workflow. In this context, it is of high relevance whether the quality and long-term performance of the restoration is influenced by the selected material [4–9]. In addition to biocompatibility, aesthetic value, and fracture stability, particularly the aspect of marginal accuracy has been described to be essential for the long-term success of prosthetic restorations [10]. The metal-ceramic mentioned above and all-ceramic materials have sufficient mechanical properties and proven biocompatibility. Therefore, the decisive factors in the CAD/CAM workflow are the precision of fit and marginal accuracy [10].

Marginal discrepancies may challenge the survival rate by causing tooth sensitivity and later a washout of the luting agent [10, 11]. It is proposed by conventional clinical wisdom that marginal imperfections can lead to recurrent caries and premature failure of the restoration [1, 2]. Microleakage through the dentinal tubules toward the pulp chamber may lead to pulpitis and the need for endodontic treatment. Furthermore, an ill-fitting restoration can cause internal stress in the restorative material and thus reduce its strength, promoting material fractures/veneering ceramic fractures and catastrophic failures of the all-ceramic framework [2]. Moreover, it is a commonly accepted clinical dogma that crowns with imperfect margins (gaps; over- or undercontoured margins) can lead to the initiation or progression of periodontal disease [12].

There are variable definitions regarding a clinically acceptable margin [6], and the available literature offers no defined threshold regarding the maximum marginal discrepancy that is clinically acceptable [13, 14]. Many authors accept the criterion established by McLean and von Fraunhofer (1971) who proposed a maximum marginal gap of 120 *μ*m after a 5-year examination of 1,000 restoration gaps [15].

The topic of the marginal accuracy of metal-ceramic and all-ceramic restorations is heavily investigated but shows some inherent limitations. In a literature review based on 183 studies, marginal gap values ranging from 7.6 to 206.3 *μ*m were identified. The outcome variations can be attributed to heterogeneous study designs with varying definitions of the marginal, direct, and indirect evaluation methods, measurements per specimen, sample size, finish line, and the stage at which the marginal gap was measured [11]. In a systematic review focused on the marginal adaptation of ceramic crowns, the marginal gap values between 3.7 and 174 *μ*m were identified [10]. The selected 54 articles showed a significant heterogeneity regarding study design, which leads to a wide range of marginal gap values, even for the same ceramic system. Therefore, it was impossible to compare results from different studies and provide a ranking of the different crown systems [10]. Consequently, for the analysis of parameters affecting the achievable marginal accuracy, only within-study comparisons are suitable.

There is no consensus on the best methodology for evaluating the fitting accuracy of prosthetic restorations. Nevertheless, based on the findings of the reviews mentioned above, a number of aspects should be addressed for improving the design quality of a comparative study on the fitting accuracy [11]. In addition to using the same measuring method (preferably on the abutment tooth or master die), the number of influencing parameters must be controlled. The measurement should be carried out on the same standardized tooth type with the same preparation design, finish line configuration, and the same cement space setting. As cementation or porcelain firing can significantly affect the marginal adaptation, all measurements have to be carried out in the same stage [16–19]. Based on the findings of Groten et al. (2000), the number of measurements per specimen should be increased as much as possible because a large number of measurements (at least 20–25) lead to more consistent distribution of the data with small standard deviations, thus improving the strength of the statistical analysis [20].

The marginal accuracy in the “as-machined state” is of interest, particularly when testing the marginal quality of a CAD/CAM system. Under these preconditions, the internal surface of the copings should not be adjusted manually [10, 11]. As documented in the literature, the process of porcelain firing can affect the fitting accuracy. Regarding the evaluation of the manufacturing quality of a CAD/CAM system, it is preferable to measure the copings before the veneering process [11, 16, 17].

More recently, several comparative studies with standardized designs investigated the influence of CAD/CAM milling machines or scanning units on the marginal accuracy [13, 21–25]. Comparative in vitro studies evaluating a possible influence of the selected restorative material on the performance of an up-to-date CAD/CAM system are still limited [8, 9, 26]. The purpose of the present study was to investigate the marginal fit of YSZ and glass-ceramic copings in comparison to cp-titanium and cobalt-chromium copings of identical design. All copings were produced in an identical digital workflow (identical master die, scanning unit, and design software) and a material specific CAM process. Differences in the production process were related to the material properties. Therefore it was not possible to use the same milling or grinding process for the different materials tested in the present study. Cobalt-chromium and titanium were processed in a milling process with tungsten-carbide instruments under constant water cooling. Zirconia was dry-milled using tungsten-carbide instruments as well. Glass-ceramics require the usage of diamond milling instruments with constant water cooling. With the CAD/CAM system used in the present study, cobalt-chromium, titanium, and zirconia were processed in a 4-axis module; the glass-ceramic was processed in a 5-axis module of the same system. All specimens were analyzed with an identical measuring technique. Due to the postulated importance of the marginal gap for a restoration's clinical success, both mean and maximum values for each material group were compared assuming that the one spot with the highest gap width determines the clinical risk of a dental restoration [13]. The null hypothesis was that there would be no differences regarding the mean marginal gap and average maximum gap values in association with the materials used.

## 2. Materials and Methods

### 2.1. Fabrication of the Experimental Model

**Figure 1:**
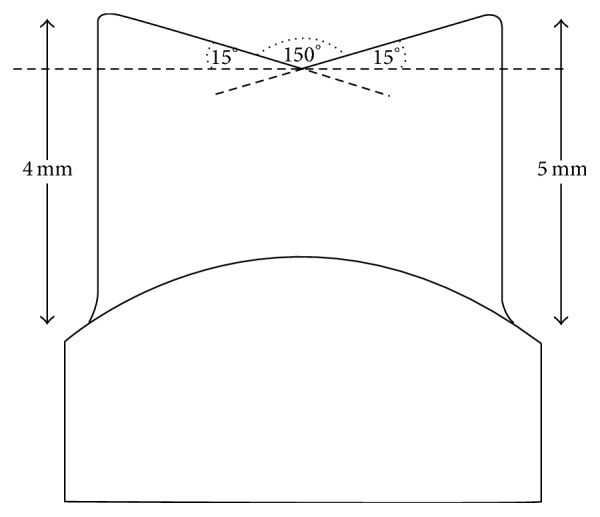
Abutment geometry.

**Figure 2:**
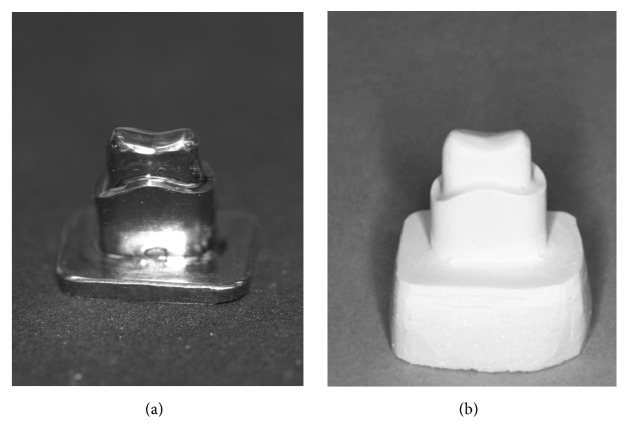
Metal master die (a) and master stone model (b).

An upper left second premolar acrylic tooth model (Frasaco, Tettnang, Germany) was prepared for a single crown with a 360° shoulder (with internal rounded line angle) and a cutting depth of 1 mm. The occlusal reduction was at least 1.5 mm, and the resulting convergence angle was set at 2 × 2° (4-degree taper) [13] (Figure 1). After impression taking, a die of casting wax was sprued, invested, and cast from a silver-palladium alloy (Palliag M, Dentsply Sirona Prosthetics, Hanau, Germany). The finished master die was used as a template for 20 master models, which were fabricated out of Type IV dental stone (GC Fujirock EP, GC Europe N.V., Leuven, Belgium) after taking impression with a polyether material (Impregum Penta, 3M Espe AG, Seefeld, Germany), simulating the clinical workflow (Figure 2).

### 2.2. Pilot Study

A pilot study was conducted to determine the suitable design parameters to achieve the optimum fit. In the CAD/CAM system used in the present study, two design parameters, which directly affect the fitting accuracy, can be selected by the operator: “cement gap” and “distance of the cement gap to the finishing line.” Ten out of the 20 fabricated working dies were used for this pilot study, applying the following 5 combinations of relevant parameters: A: cement gap 30 *μ*m and distance to finish line: 1 mm (this was the factory default setting); B: cement gap 30 *μ*m and distance to finish line: 1.5 mm; C: cement gap 40 *μ*m and distance to finish line: 1 mm; D: cement gap 40 *μ*m and distance to finish line: 1.5 mm; and E: cement gap 40 *μ*m and distance to finish line: 0.5 mm.

For groups B–E, two copings of each material were fabricated and evaluated for the marginal fitting accuracy in the “as machine” state. The best fitting quality for all materials was achieved with the parameters of group B (cement gap = 30 *μ*m, distance cement gap to finish line: 1.5 mm).

### 2.3. Main Study

**Figure 3:**
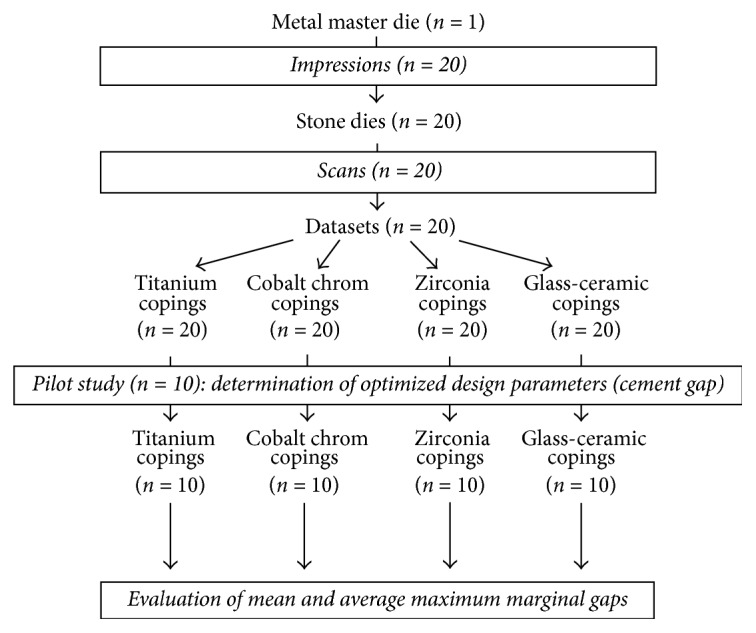
Study design.

The remaining 10 working designs were used to fabricate 4 copings of the four different materials included in the present study,

- commercially pure- (cp-) titanium grade IV (DENTAURUM GmbH & Co. KG, Ispringen, Germany),
- cobalt-chromium alloy (CoCr) type 4 (Kera-Disc, Eisenbacher Dentalwaren ED Inc., Woerth am Main, Germany),
- yttria-stabilized presintered zirconia (YSZ) (Z-CAD, METOXIT AG, Thayngen, Switzerland),
- leucite-reinforced glass-ceramic (IPS Empress CAD, Ivoclar Vivadent, Schaan, Liechtenstein),

with optimized design parameters determined in the pilot study (cement gap = 30 *μ*m; distance cement gap to finish line = 1.5 mm). Forty copings were evaluated for the mean and average maximum marginal gaps at 24 points of measurement per specimen, resulting in 960 measurements (Figure 3).

### 2.4. Fabrication of the Restorations

The process of fabricating the different copings consisted of scanning and digitizing the working dies with a lab-based scanner (OpenScan 100, Laserdenta, Bergheim, Germany), designing the crown copings with a uniform thickness of 0.7 mm using a CAD software (OpenCAD V.4, Laserdenta, Bergheim, Germany), and manufacturing the optimized designs with the corresponding 5-axis milling machine (GAMMA 202, Wissner GmbH, Goettingen, Germany). Each working die was scanned once, and a coping was designed. This CAD design was applied to all four materials. Titanium, cobalt-chromium alloy, and YSZ were milled in a 3-axis milling module, while the glass-ceramic blocks had to be ground in a 5-axis system with constant water cooling. The manufacturing process of the YSZ copings was finished by a high-temperature sintering process (1,350°C, 8 h). The milled glass-ceramic copings were finalized by a 10-minute crystallization firing at 850°C.

### 2.5. Measurement of the Marginal Fit

**Figure 4:**
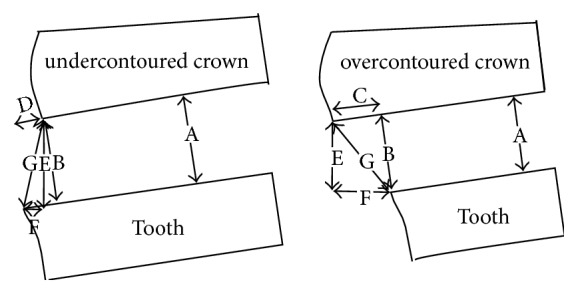
Definition of the cervical marginal gap (according to Holmes et al. 1989): A = internal gap; B = marginal gap (measured in the current study); C = overextended margin; D = underextended margin; E = vertical marginal discrepancy; F = horizontal marginal discrepancy; G = absolute marginal discrepancy.

After finishing the fabrication process, the marginal fit of the copings was evaluated in the “as-machined state” using the identical measuring technique as published earlier [13]. To ensure the comparability of the machined copings, no specimen was manually adapted internally or finished on the outside contour. The cervical marginal gap was defined according to Holmes et al. 1989 as the discrepancy between the finish line (tooth) and the crown margin at an angle of 90 degrees to the crown margin (Figure 4) [14].

**Figure 5:**
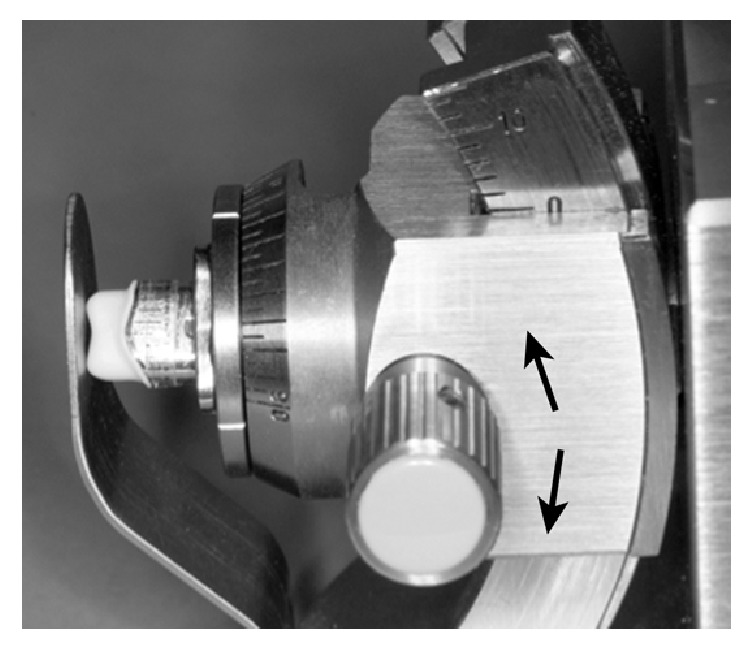
Pivoted socket with fixed coping.

**Figure 6:**
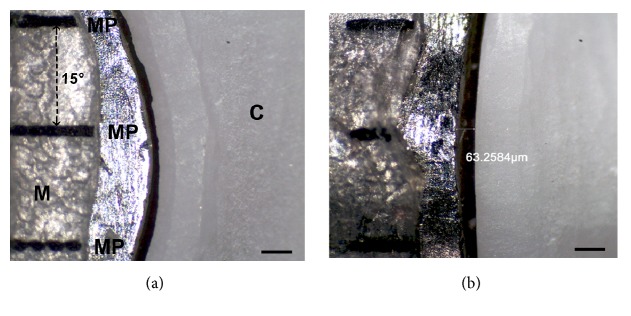
Images of the marginal gap: (a) measuring points (MP), master die (M) and coping (C); (b) measured with Adobe Photoshop Software.

Each coping extent was divided into 24 equal sections shifted by 15° scaled around the master die as published earlier [13] and in accordance with the criteria established by Groten et al. [20]. The master metal die with the coping was fixed in a specially designed device for fixing the coping with a constant exerted pressure ensured by the use of a tension spring (Figure 5) [13]. Furthermore, this device had a pivoted socket to ensure a continual accurate vertical optical axis for assessment of the marginal gap (represented by line segment “B = marginal gap” in Figure 4). Therefore over- or underextended margins have no effect. The marginal gap was assessed using photographical images of all 24 measured points taken by a Leica EZ4 D microscope with integrated camera (Leica-Microsystems, Wetzlar, Germany) in an angle of 90 degrees to the marginal gap. The images were evaluated using the measurement tool of the Adobe Photoshop CS5 Software (Adobe Systems Incorporated, San José, USA) (Figure 6).

### 2.6. Statistical Analysis

The sample size calculation was based on the mean and SD, according to Brawek et al. (2013) [27]. The sample of 10 specimens (each with 24 points of measurement) for each group achieved a 79% power to detect differences among the mean values, with a 0.05 (*α*) significance level.

The assumption of normality of the data was checked by using the Kolmogorov-Smirnov test and parametric test was selected for the further statistical analysis.

The mean value for the marginal gap was evaluated for each material. The maximum marginal gap of each coping was used to calculate an averaged maximum marginal gap value for each material group. The mean marginal gap values and averaged maximum gap values were analyzed using one factor repeated measures-design with the factor “materials” and factor subgroups “titanium,” “cobalt-chromium,” “YSZ,” and “glass-ceramic” at a significance level of *α* = 0.05. *p* values of the pairwise comparisons were then adjusted by using the Bonferroni-Holm method. All analyses were carried out using a statistical software package (SAS Version 9.3, SAS Institute Inc., Cary, NC, USA). The statistical calculation and interpretation were performed in collaboration with the Institute of Medical Statistics, University Medical Center Goettingen, Goettingen, Germany.

## 3. Results

**Figure 7:**
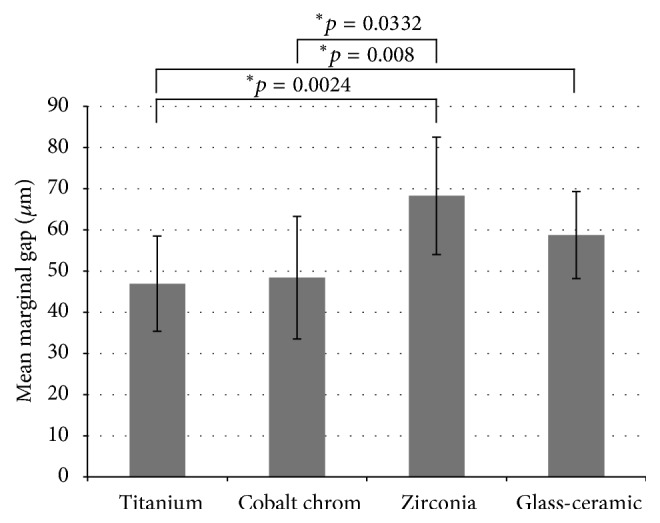
Comparison of the mean values (*μ*m) and standard deviations in precision of fit of different materials. Asterisks show significant differences between the groups.

The mean marginal gaps ranged from 46.92 ± 23.12 *μ*m for titanium copings to 68.25 ± 28.54 *μ*m for YSZ copings (Table 1). The difference in the mean marginal gaps comparing titanium/YSZ (*p*adj = 0.0024), titanium/glass-ceramic (*p*adj = 0.008), and cobalt-chromium/YSZ (*p*adj = 0.0332) was statistically significant (Figure 7). The mean marginal gap for the cobalt-chromium coping (48.37 ± 29.72 *μ*m) was not significantly different from the respective values determined for the titanium chromium (46.92 ± 23.12 *μ*m) and the glass-ceramic coping (58.73 ± 21.15 *μ*m).

The averaged mean maximum values ranged from 91.54 ± 23.39 *μ*m (titanium) to 120.66 ± 32.75 *μ*m (YSZ). There were no statistically significant differences among the four analyzed groups (*p* = 0.1673) regarding the average maximum values of marginal discrepancy (Table 1).

For the parameter maximum marginal gap, the lowest value was determined for group D (glass-ceramic) = 118.03 *μ*m, followed by group A (titanium) = 143.71 *μ*m. Similar to the other two parameters, the highest value was determined for group C (YSZ) = 183.15 *μ*m (Table 1).

Only for the coping from group D, all measured marginal gap values in the “as-machined state” were within the level of clinical acceptable fitting quality (<120 *μ*m according to McLean and von Fraunhofer 1971).

## 4. Discussion

In this in vitro study, the use of a metal die as the single standard master die and the ensuring of a consistent process chain allowed direct comparison discrepancies in marginal fit between four different restorative materials. The mean marginal gap values for the four different materials range from 46.92 ± 23.12 *μ*m (titanium) to 68.25 ± 28.54 *μ*m (YSZ), demonstrating a significant difference. This effect could not be detected for the parameter average maximum marginal gap. Nevertheless, an association of the selected restorative material on the fitting accuracy in the as-machined state could be demonstrated. Therefore, the null hypothesis has to be partially rejected.

The threshold for an acceptable marginal discrepancy remains without clinical or evidence-based consensus. Nevertheless, there is a consensus that a marginal gap of less than 120 *μ*m is clinically reasonable (McLean and Fraunhofer 1971). In this study, all mean values were within clinically acceptable limits. Moreover, it could be demonstrated that CAD/CAM-fabricated crown copings of a specific material (glass-ceramic) in the “as-machined state” already offer a marginal fitting quality that fulfills the level of clinical acceptability (all marginal gap values < 120 *μ*m).

Based on the a priori power analysis, a type II error of *β* = .20 was accepted. By using an increased number of specimens, the power of the performed statistical analysis could have been increased. This might be a relevant limitation of the present study. Particularly the statistical analysis of the mean maximum marginal gap values might be limited by the reduced number of observations. During analysis of these data, however, a difference in the mean values of more than 29 *μ*m did not result in a significant difference.

For this scientific investigation, an in vitro evaluation method for the marginal fit of CAD/CAM-manufactured copings was selected. The main target was to evaluate a potential effect of the restorative materials and their specific CAM process on the initial fitting accuracy in the “as-machined state.” The selected direct view technique using a light microscope is most commonly used for such in vitro investigations. The direct view technique offers the advantage of not incorporating any procedures on the crown-die assembly (sectioning or replica-technique), thus reducing the chance of error accumulation from a multistep procedure. The limits of the technique are related to the selection of the points of measurement for the marginal gap, as margins of the crown or the preparation may appear rounded. Furthermore, the direct microscopic examination of the marginal fit is limited by projection errors [10, 11]. To minimize these errors, the affixation of the specimens 90° to the optical axis of the light microscope is very important. This precondition was fulfilled by the customized holding device used in the present study [13]. The copings were manufactured based on the same master die using an identical fabricating process chain. The fitting quality was evaluated on the master die representing the abutment tooth. Therefore, the comparability of all measurement series is guaranteed. All copings were produced and tested under nearly ideal conditions. These aspects are imperative for a comparative evaluation of the fitting accuracy [10, 11, 13]. For the interpretation of the findings of this in vitro study, it is important to recall that the findings report the fitting quality in the “as-machined state.” On the one hand, the fitting quality might be positively affected by a manual adjustment of the coping [11]. On the other hand, such grinding procedures are a source of distortion and should not be used if the effects of a manufacturing technique or the restorative material need to be evaluated. Furthermore, the fitting accuracy was evaluated in the coping stage. Therefore, the marginal gap values of the present study do not represent the values achievable under clinical conditions. The final fitting quality of the crown can be affected by subsequent fabrication (manual adaptation) and clinical procedures (porcelain firing, cementation) [16–19]. Moreover, the fact that confounding factors such as the patient's compliance, wet oral environment, and limitation of vision were eliminated in the present study design should be considered [10, 11].

Therefore, the design of the present study primarily allows the evaluation of a possible association of the selected restorative material on the fitting quality in the “as-machined state” rather than a detailed conclusion of the achievable fitting accuracy in clinical settings.

The fitting accuracy of indirect restorations has been evaluated in numerous in vitro and in vivo trials. However, various methods for measuring and evaluating the marginal gap are described [10, 11, 14, 15]. These methodological differences make it challenging to compare the results from different studies. For example, the marginal gap values for glass-infiltrated aluminous core restorations (InCeram, Vita Zahnfabrik Bad Säckingen, Germany) reported in the literature range from 7.5 to 161 *μ*m [11]. However, considering the results of studies using a similar study design and identical evaluation, the reported values for the marginal gap range from 49.8 to 57 *μ*m [28–30]. Therefore, it is vital to compare the results of the present study to findings of studies using the same study design.

Rinke et al. 2012 reported results from an in vitro trial using the identical geometry of the master die and the identical measuring technique (direct view, light microscope, 24 points of measurement per specimen). They reported the results for the mean marginal gap and the averaged maximum gap of zirconia copings in the “as-machined state” and after manual adaptation [13]. The mean marginal gap values for zirconia copings in the “as-machined state” fabricated by different CAD/CAM systems ranged from 57.94 ± 6.5 *μ*m to 71.01 ± 10.8 *μ*m. For the parameter averaged maximum marginal gap value, they ranged from 121.03 ± 19.2 *μ*m to 114.6 ± 30.5 *μ*m. This finding is in good accordance with the findings of the present study (mean marginal gap: 68.25 ± 28.54 *μ*m, averaged maximum marginal gap: 120.66 ± 32.75 *μ*m).

A comparison of the findings of the present study with the above-mentioned study indicated that the CAD/CAM system used in the present study allows the fabrication of zirconia copings, as this CAD/CAM system is well established in the dental field for 10 years.

Based on the findings of the present study, the performance of the specific CAD/CAM system used in the present study in relation to the marginal adaptation is influenced by the restorative material. This result is well in line with the findings of another comparative in vitro study using a different CAD/CAM system. In an in vitro study using the KaVo Everest CAD/CAM system, no significant difference in the marginal gap values of YSZ (58.6 ± 4.4 *μ*m) and glass-ceramic crowns (54.7 ± 9.4 *μ*m) was detected. In the present study, the lowest marginal gap values were detected for titanium copings (18.3 ± 3.4 *μ*m) [4].

In another comparative in vitro study, the marginal gap values for lithium-disilicate crowns were reported to be significantly smaller than for YSZ crowns [5]. Furthermore, the effect of the restorative material on the marginal accuracy was also reported for FDPs and implant-supported FDPs in comparative in vitro studies. Two comparative studies reported statistically significant smaller marginal gap values for CAD/CAM-fabricated CoCr and titanium implant-retained FDPs compared to FDPs milled from partially sintered YSZ [6, 7]. Two other studies reported smaller marginal gap values for FPDs fabricated from a CoCr alloy compared with YSZ FDPs [8, 9].

In all these studies, a superior fitting accuracy of the CAD/CAM-fabricated metallic specimens (CoCr alloy and cp-titanium) compared with YSZ specimens was reported [4–9]. This result is in agreement with the findings of the present study, revealing significantly higher mean marginal gap values for YSZ crown copings compared with crown copings fabricated from a CoCr alloy (group B) or cp-titanium (group A).

A possible explanation for the improved overall fitting quality of the two groups (A, B) of metallic copings compared with the two all-ceramic materials can be seen in the sintering and crystallization process required to bring these materials to their final density and strength [3]. Zirconia is milled in a presintered stage, and a sintering process is needed to bring the material to its final density. The sintering process leads to volumetric changes in the materials. This shrinkage of the material has to be compensated by milling the restoration in an enlarged state [13]. Compared to the metallic materials that are milled in their final state, the sintering process introduces an additional source of error that can affect the marginal accuracy at least in the “as-machined state.” To a lesser extent, the crystallization process is related to a dimensional change and therefore can represent a potential source of error.

Another potential influencing factor might be seen in the CAM procedure used. All specimens were produced with the identical milling machine but with different processing routes. The metallic copings were fabricated in a high-speed milling process using tungsten-carbide instruments under constant water cooling. For the all-ceramic materials, two different procedures were applied. Zirconia was milled in a dry state, and the glass-ceramic material was ground using diamond-coated instruments [3]. These differences are related to the material properties of the different materials, and it is not possible to use the same CAM process for the four materials evaluated in the present study. Although the same milling unit was used, the differences in the processing routes (milling versus grinding) and differences in the instruments used (tungsten-carbide versus diamonds) might be an influencing factor for the achievable marginal accuracy. The types of instruments and their difference in wear might additionally affect the achievable fitting accuracy. Furthermore, it could be demonstrated that ceramic materials are more prone to material fractures (chipping) during the production process than alloys or metals and composite materials [31].

## 5. Conclusion

Considering the limitations of the study, the following conclusions were drawn:

- CAD/CAM-fabricated crown copings from different materials (cp-titanium, CoCr alloy, YSZ, and glass-ceramic) reach mean marginal and averaged maximum marginal gap values well within the clinically acceptable marginal gap range (<120 *μ*m).
- The achievable marginal accuracy of CAD/CAM-fabricated crowns is significantly influenced by the restorative material. YSZ copings showed significantly increased mean and averaged maximum marginal gap values compared to the CoCr and cp-titanium copings.

## Figures and Tables

**Table 1:** Values of the mean and averaged maximum and maximum marginal gaps of each material (in *μ*m).

Material	Mean marginal gap	SD (*σ*)	Averaged maximum marginal gap	SD (*σ*)	Maximum marginal gap
Titanium	46.92	23.12	91.54	23.39	143.71
Cobalt-chromium	48.37	29.72	96.86	24.19	156.44
YSZ	68.25	28.54	120.66	32.75	183.15
Glass-ceramic	58.73	21.15	100.22	10.83	118.03

Marginal fitting of the copings.
